# Establishment of Recombinant Trisegmented Mopeia Virus Expressing Two Reporter Genes for Screening of Mammarenavirus Inhibitors

**DOI:** 10.3390/v14091869

**Published:** 2022-08-25

**Authors:** Lisa Oestereich, Stephanie Wurr, Beate Becker-Ziaja, Sabrina Bockholt, Meike Pahlmann, Daniel Cadar, Beate M. Kümmerer, Stephan Günther, Romy Kerber

**Affiliations:** 1 Bernhard Nocht Institute for Tropical Medicine, 20359 Hamburg, Germany; 2 German Center for Infectious Research (DZIF), Partner Site Hamburg-Lübeck-Borstel-Riems, Partner Side Hamburg, 20359 Hamburg, Germany; 3 Institute of Virology, University of Bonn Medical Centre, 53127 Bonn, Germany; 4 German Center for Infection Research (DZIF), Partner Site Bonn-Cologne, 53127 Bonn, Germany

**Keywords:** negative-strand RNA virus, mammarenavirus, reverse genetics, antivirals, Mopeia virus

## Abstract

Highly pathogenic Arenaviruses, like the Lassa Virus (LASV), pose a serious public health threat in affected countries. Research and development of vaccines and therapeutics are urgently needed but hampered by the necessity to handle these pathogens under biosafety level 4 conditions. These containment restrictions make large-scale screens of antiviral compounds difficult. Therefore, the Mopeia virus (MOPV), closely related to LASV, is often used as an apathogenic surrogate virus. We established for the first time trisegmented MOPVs (r3MOPV) with duplicated S segments, in which one of the viral genes was replaced by the reporter genes ZsGreen (ZsG) or Renilla Luciferase (Rluc), respectively. In vitro characterization of the two trisegmented viruses (r3MOPV ZsG/Rluc and r3MOPV Rluc/ZsG), showed comparable growth behavior to the wild type virus and the expression of the reporter genes correlated well with viral titer. We used the reporter viruses in a proof-of-principle in vitro study to evaluate the antiviral activity of two well characterized drugs. IC_50_ values obtained by Rluc measurement were similar to those obtained by virus titers. ZsG expression was also suitable to evaluate antiviral effects. The trisegmented MOPVs described here provide a versatile and valuable basis for rapid high throughput screening of broadly reactive antiviral compounds against arenaviruses under BSL-2 conditions.

## 1. Introduction

Emerging viruses such as Ebola (EBOV), Marburg (MARV), Nipah, SARS-CoV-2, or Lassa virus (LASV) have caused several outbreaks in recent years and pose a serious threat to public health in the affected countries [1]. Zoonotic RNA viruses are responsible for more than 75% of newly discovered or re-emerging diseases [2], and the ongoing SARS-CoV-2 pandemic and the outbreaks of EBOV and MARV in the Democratic Republic of Congo, Guinea, and Ghana in 2021 and 2022 emphasize the importance of research and development for these pathogens [3,4,5]. Besides vaccines and diagnostics, broadly acting therapeutics and vaccines are urgently needed to help combat these viruses [6]. Especially the development of broad-spectrum antivirals would allow fast reactions to outbreaks of known, but also closely related new viruses.

Mammarenaviruses, like LASV, are predominantly rodent-born enveloped negative-stranded RNA viruses that occur worldwide, with more than 40 distinct species as of 2018 [7]. Of those, seven are highly pathogenic and can cause hemorrhagic fever in humans. These are the New World arenaviruses Junín (JUNV), Guanaritio, Machupo, Sabia, and Chapare that occur in South America, whereas the Old World arenaviruses LASV and Lujo (LUJV) can be found in Africa [8]. LASV is endemic in Guinea, Sierra Leone, Liberia, and Nigeria [9], and based on seroprevalence studies it is estimated that up to 300,000 people are infected annually, with 5000 deaths, imposing major public health and economic burdens on the affected countries [10]. Infection prevention and treatment options remain limited. Only the off-label use of Ribavirin against LASV infection is available for treatment [11]. However, the overall efficacy of Ribavirin remains a matter of contention, and a benefit was only seen if given early after the onset of disease [12,13].

Research and development (R&D) for the highly pathogenic LASV has been hampered by the requirement of biosafety level 4 laboratories for the handling of infectious material, which restricts R&D to a few laboratories worldwide. Moreover, most of these laboratories are not equipped for large-scale antiviral screening. However, advances in molecular biology and the establishment of reverse genetic systems allow the generation of reporter gene-containing recombinant arenaviruses for mid to high throughput screening (HTS) of antiviral compounds. The advantage of recombinant viruses versus surrogate models such as replicon systems, which only emulate virus transcription and replication [14,15,16], is that drugs targeting any step of the viral lifecycle can be identified and characterized. The arenavirus genome is bisegmented and consists of a small (S) and a large (L) RNA segment with an ambisense coding strategy. The S segment encodes the nucleoprotein (NP) and the glycoprotein precursor, whereas the L segment encodes the small zinc-binding matrix protein Z and the large L protein [17,18]. For segmented RNA viruses with ambisense coding strategy, two promising approaches for the generation of reporter gene-expressing recombinant viruses have been successfully realized and published. Bisegmented bicistronic viruses with additional open reading frames (ORFs) in the S segment have been reported for Lymphocytic Choriomeningitis virus (LCMV), LASV, LUJV, and Tacaribe virus (TACV) [19,20,21,22,23,24]. In most of these viruses, the ORF for the nucleoprotein (NP) was replaced by a bicistronic ORF encoding for green fluorescent protein (GFP), P2A, and the respective NP. The self-cleaving P2A peptide is derived from porcine teschovirus-1 and leads to “ribosomal skipping”, and thus to the production of NP and GFP from one transcript. In the trisegmented approach, the S segment is duplicated and one of the viral genes on each segment is replaced by a foreign gene of interest, such as reporter genes or model antigens. As of now, recombinant trisegmented LCMV expressing GFP and chloramphenicol acetyltransferase (CAT), Gaussia (Gluc) or firefly luciferase (Fluc) [25,26,27], a JUNV, and a TACV expressing GFP and Gluc [22,27], as well as a Pichinde virus (PICV) with GFP and influenza virus proteins as model antigens [28,29] have been developed. The trisegmented arenaviruses are usually genetically stable and show no or only moderate attenuation in in vitro assays, making them suitable for HTS, and facilitating the identification of antiviral compounds [25].

Mopeia virus (MOPV), a non-pathogenic mammarenavirus, closely related to LASV, is often used as a surrogate model virus for LASV. Due to its classification as a risk group 2 pathogen, MOPV-based reporter gene-expressing recombinant viruses are suitable for a variety of HTS facilities without needing special biosafety conditions.

In this study, we described a reverse genetic system for the generation of recombinant bi- and trisegmented MOPV. We rescued and characterized wild type (r2MOPV wt) and bicistronic Renilla luciferase (Rluc) expressing MOPV (r2MOPV Rluc), as well as two different trisegmented (r3MOPV) viruses with ZsGreen (ZsG) and Rluc. Moreover, we established a system suitable for mid- to high-throughput screens for antiviral compounds using the Rluc-expressing r3MOPV for future identification of novel antiviral compounds against mammarenaviruses.

## 2. Materials and Methods

### 2.1. Cells and Viruses

All virus amplifications, growth curves, and antiviral kinetics were performed on Vero E6 cells subclone FM (henceforth called Vero FM) [30]. Virus rescue was done in low passage (<P 50) baby hamster kidney (BHK-21/J) [31] or BSR-T7/5 cells which stably express T7 RNA polymerase [32]. Vero FM and BHK cells were maintained in Dulbecco’s minimal essential medium (DMEM) supplemented with 5% fetal calf serum (FCS), 100 U/mL penicillin, 100 µg/mL streptomycin, 1 mM glutamine, 0.5 mM pyruvate, and 1x non-essential amino acids (all from Pan Biotech, Aidenbach, Germany) at 37 °C and 5% CO_2_. BSR-T7/5 cells were grown in Glasgow’s minimal essential medium (GMEM; GIBCO/Thermo Fisher Scientific, Waltham, MA, USA) with 5% FCS, and 1 mg/mL Geneticin (GIBCO) was added to the cells every second passage to maintain the T7 polymerase transgene.

MOPV strain AN 21366-BNI (GenBank accession numbers JN561684 and JN561685; seed stock obtained from National Collection of Pathogenic Viruses, Porton Down, Salisbury, UK) was propagated, and directly sequenced, including the conserved S and L RNA termini as described previously [14,33]. Low passage (<5 passages), wild type MOPV was used for all experiments. Recombinant viruses were passaged twice on Vero FM cells (infection with MOI 0.01) before being used for further experiments.

### 2.2. Plasmids 

**Figure 1:**
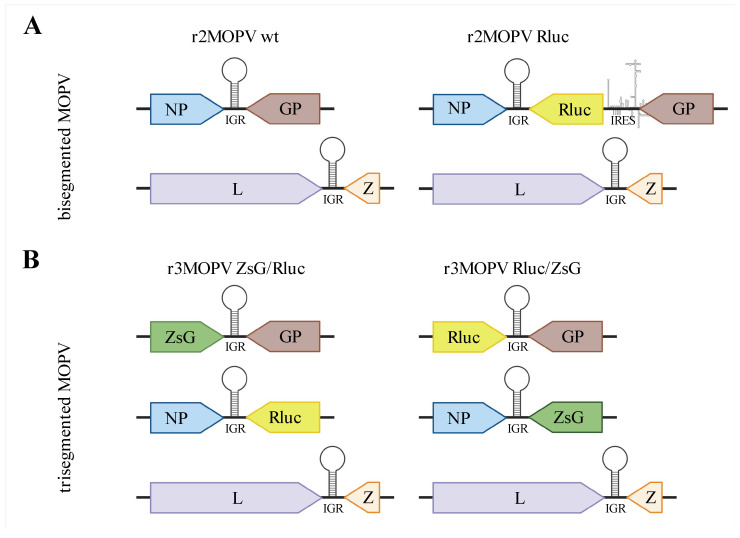
Overview of bi- and trisegmented MOPV genomes. (**A**) bisegmented MOPV containing wild type L and S segments or a modified S segment with a GP-IRES-Renilla luciferase (Rluc) ORF. (**B**) trisegmented MOPV containing two modified S segments each; either NP or GP have been replaced by Rluc or ZsGreen (ZsG) on the segment. The figure was created with BioRender.

For the expression of MOPV NP and L protein, coding sequences for both genes were cloned into the vectors pCITE 2a (Novagen/Merck KGaA, Darmstadt, Germany) (T7 polymerase-based expression) and pCAGGS (Pol-II-based expression) [33,34,35]. The full viral S and L genome segments, both in genomic and antigenomic orientation, were cloned either into the T7 polymerase-driven pX12ΔT vector [32] or the Pol-I-driven pDP vector [34,36]. Pol-I-promotor-based modified S segments were cloned to produce reporter gene expressing viruses. For the bisegmented Rluc-expressing recombinant MOPV (r2MOPV Rluc), GP was replaced by the bicistronic GP-IRES-Rluc ORF. To that end, the N-terminus of GP was fused to an internal ribosome entry site (IRES) followed by Rluc. For trisegmented ZsG and Rluc-expressing MOPV (r3MOPV ZsG/Rluc and r3MOPV Rluc/ZsG) either NP or the glycoprotein GP were replaced by the coding sequence for ZsG or Rluc. An overview of all produced plasmids can be found in Figure 1. All modified S segments were cloned in antigenomic orientation into the pDP vector. Final S and L genome sequences matched the consensus sequence except for an A to G exchange at position 163 in the GP gene, leading to an amino acid change from isoleucine to valine in the plasmids r3MOPV ZsG/GP and r3MOPV Rluc/GP.

### 2.3. Rescue of Recombinant Virus

For the rescue of rMOPV starting from T7 promotor-driven plasmids, BSR-T7/5 cells were seeded at a density of 1 × 10^5^ cells/well in 6-well plates one day before transfection. Cells were transfected with Lipofectamine^TM^ 2000 (Life Technologies, Carlsbad, CA, USA) at a Lipofectamine/DNA ratio of 3 µL per 1 µg with 1500 ng L-pCITE, 750 ng NP-pCITE, 750 ng S-pX12ΔT, and 1500 ng L-pX12ΔT according to the manufacturer’s protocol. For negative control, the L segment was replaced by an empty pX12ΔT vector. BHK-J cells for the Pol-I/II-driven rescues were seeded at a density of 1.2−1.5 × 10^5^ cells/well in 6-well plates one day before transfection. Amounts of 1500 ng L-pCAGGS, 750 ng NP-pCAGGS, 750 ng S-pDP (wt or modified), and 1500 ng L-pDP or empty vector were transfected into the cells using Lipofectamine at a ratio of 3:1, analogous to the transfection of BSR-T7/5 cells. For the rescue of r3MOPV, two S segment-containing vectors (750 ng each) were transfected (see Figure 1).

The transfection mixture was removed 4 h post transfection, and growth medium containing 5% FCS was added to the cells. Cells were transferred to T-25 flasks upon reaching confluence, followed by a stepwise transfer to T-75 and T-175 flasks. Cells were further split 1:10 every 3–4 days for three weeks. At each cell transfer, an aliquot of cells was spotted on microscopy slides for immunofluorescence staining, and 1 mL of the cell supernatant was stored at −80 °C for virus quantification. Successfully rescued viruses were amplified on Vero FM cells to generate experimental stocks and sequenced by Sanger sequencing to confirm the absence of mutations.

### 2.4. Virus Quantification and Immunofluorescence Microscopy

Virus quantification was done by immunofocus assay as described previously [37] or by RT-PCR. For RT-PCR, viral RNA from cell culture supernatant was extracted using the QIAamp Viral RNA Mini Kit (Qiagen, Hilden, Germany) according to the manufacturer’s instructions. The QuantiTect SYBRgreen RT-PCR kit from Qiagen with MOPV-specific primers MO_S_2386-fwd (GATCGATGTTCTTGATGCTATG) and MO_S_2610-rev (GTGAAAGCTGGTGCTTGTCTA) were used. The RT-PCR reaction was performed in a LightCycler 2.0 (Roche, Basel, Switzerland) using the following temperature profile: 50 °C for 20 min, 95 °C for 15 min, followed by 44 cycles of 94 °C for 15 s, 55 °C for 20 s, and 72 °C for 30 s.

To assess the rescue success of recombinant viruses using immunofluorescence microscopy, cells were trypsinized, washed in PBS, spotted on epoxy-coated microscopy slides, and fixed in acetone [38]. Cells transfected with plasmids for the rescue of r2MOPV wt, r2MOPV Rluc, or negative controls were stained with the Old World arenavirus NP-specific monoclonal antibody 2LD9 [39], followed by a FITC-conjugated secondary antibody. Cells transfected for the rescue of fluorescence protein expressing r3MOPV variants were directly used for microscopy after fixation. For the growth kinetics and the antiviral activity assays, Vero FM cells were directly seeded on 7 mm diameter glass slides in 24-well plates. Infection of cells was performed as described above. After the removal of medium (days 1–3 for the growth kinetics and day 3 for the antiviral assays), cells were washed once with PBS and fixed for 30 min at room temperature by the addition of 4% formaldehyde in PBS. Cells were permeabilized with 0.5% Triton-X-100 in PBS for 20 min and stained for NP with the antibody 2LD9, and a secondary antibody labeled with Alexa 568 (Life Technologies, Carlsbad, CA, USA). Nuclei were stained with DAPI, and slides were mounted on microscopy slides with Mowiol. All slides were imaged with a fluorescence microscope Axio Imager M1 (Zeiss, Oberkochen, Germany).

### 2.5. Measurement of Luciferase Activity

Luciferase activity in infected cells was determined using the Renilla Luciferase Assay System (Promega; Madison, WI, USA) according to the manufacturer’s instructions. In short, cells were washed with PBS and lysed by adding 100 µL Renilla Luciferase Assay Lysis Buffer (Promega) per well of a 24-well plate. After 20 min incubation at room temperature on a horizontal shaker, lysed cells were transferred in 1.5 mL tubes and cell debris was centrifuged at 10,000× *g* for 1 min. Luminescence was determined by mixing 50 µL Renilla Luciferase Assay Reagent with 10 µL cell lysate and measuring the luminescence over 10 s in the Luminometer Junior LB (Berthold Technologies, Bad Wildbad, Germany) or the GloMax^®^ Navigator (Promega; Madison, WI, USA).

### 2.6. Next-Generation Sequencing of rMOPV

To determine the genetic stability of rMOPV variants, viruses were passaged ten times on Vero FM cells. For each passage, virus was harvested 3 days post infection and RNA concentrations were determined using RT-PCR. Fresh Vero FM cells were subsequently infected with 1 RNA copy/cell. Next-generation sequencing (NGS) was performed after 2, 5, and 10 passages. RNA was prepared with the QIAamp Viral RNA Mini Kit (Qiagen, Hilden, Germany) according to the manufacturer’s instructions, excluding the carrier-RNA step. The extracted viral RNA was subjected to an unbiased metagenomic approach described elsewhere [40]. Briefly, after random RT-PCR amplification of the RNA, the extracted viral RNA was subjected to library preparation using a QIAseq FX DNA Library Kit (Qiagen, Hilden, Germany). Samples were normalized by KAPA qPCR (Roche), pooled and sequenced using 200-cycle (2 × 100 bp paired-end) P2 reagent kits (Illumina, San Diego, CA, USA) on a NextSeq 2000 platform. The generated raw data was cleaned from low-quality reads and the polyclonal sequences were removed. The curated sequence data were then compared with viral databases (https://www.ncbi.nlm.nih.gov/genome/viruses/, accessed on 20 June 2020). The cut-off E-value for the BLASTx analyses and comparison was set to 0.001. The viral metagenomic output was visualized and analyzed in MEGAN (https://software-ab.informatik.uni-tuebingen.de/download/megan6/welcome.html; accessed on 30 July 2020). The reads were assembled de novo using Geneious 9 (Biomatters, Auckland, New Zealand) and CLC Workbench (Qiagen, Hilden, German), and then contigs larger than 100 bp were subjected to mapping using MOPV sequences from GenBank (NC_006575; NC_006574). 

### 2.7. Growth Kinetics

Vero FM cells were seeded at a density of 1 × 10^6^ cells per 24-well plate one day before infection. Cells were inoculated with 100 µL per well with rMOPV in DMEM without FCS, with a MOI of 0.01, and incubated for 1 h at 37 °C. Afterwards, the cell supernatant was discarded, the cells were washed, and 1 mL growth medium containing 5% FCS was added per well. At each kinetic time point cell culture supernatant samples for virus quantification were taken and immediately stored at −80 °C. Infected cells were used for immunofluorescence staining and for measurement of Rluc activity.

### 2.8. Assessment of Antiviral Activity 

Favipiravir (T-705, CAS no. 259793-96-9; BOC Sciences, Creative Dynamics, New York, NY, USA) was dissolved in DMSO, and Ribavirin (CAS no. 36791-04-5; MP Biomedical, Irvine, CA, USA) was dissolved in 0.9% NaCl solution at a concentration of 100 mM and stored at −20°C. The compounds were diluted half logarithmic from 300 µM to 1 µM in DMEM containing 5% FCS. To correct for DMSO-based effects, DMSO at a final concentration of 1% was added to all drug dilutions. Vero FM cells were seeded at a density of 1 × 10^6^ cells/24-well plate one day before they were inoculated with a MOI of 0.01. The inoculum was removed after 1 h and replaced with medium supplemented with the different concentrations of compounds. Cell culture supernatants were harvested 3 days post infection and stored at −80 °C for virus quantification. Infected cells were used for immunofluorescence staining and for measurement of Rluc activity.

### 2.9. Software and Data Analysis

Graphs were generated with GraphPad Prism 9 and the schematic figure was prepared with BioRender. Immunofluorescence microscopy pictures were edited with ImageJ. The inhibitory concentration that reduced the virus titer by 50% (IC_50_ value) was calculated with the sigmoidal 4PL function in Prism. 

## 3. Results

### 3.1. Rescue of Bisegmented MOPV

Recombinant MOPV wt (r2MOPV wt) was rescued with plasmids in genomic and antigenomic orientation in both the T7 polymerase-based system and the Pol-I/II-based system (Figure 1 and Table 1).

Rescue with the Pol-I/II-based systems were more reproducible and efficient with 80% virus rescue, independent of genomic orientation. The growth behavior of the different recombinant viruses was compared to wt MOPV, and no differences in the kinetic or maximum titers were observed (Supplementary Figure S1). All subsequent rescues were done in the Pol-I/II promotor-based system with the L and S segment in antigenomic orientation.

#### Rescue of a Bicistronic MOPV Expressing Rluc

For the generation of a bisegmented reporter virus, the GP ORF was replaced by the bicistronic GP-IRES-Rluc ORF. The resulting r2MOPV Rluc was successfully rescued and passaged once on Vero FM cells. The P1 r2MOPV Rluc virus showed similar initial growth behavior as the r2MOPV wt (Supplementary Figure S2). Virus titers peaked 4 days post infection and virus titers, genome copies, and relative light units (RLU) as a measure of Rluc activity correlated. Measurement of luciferase activity over two more passages showed a rapid loss of luciferase activity, and sequencing revealed that the virus quickly mutated back to the wild type S segment by deletion of the entire IRES-Rluc cassette.

### 3.2. Generation and Characterization of Trisegmented MOPV

As the bicistronic r2MOPV Rluc was genetically not stable, we used the trisegmentation approach to rescue reporter viruses. The rescue was successful for both versions, the r3MOPV ZsG/Rluc with ZsG in the NP ORF and Rluc in the GP ORF, and r3MOPV Rluc/ZsG with Rluc in the NP ORF and ZsG replacing the GP in the second S segment. The rescued viruses were passaged ten times on Vero FM cells to evaluate their genetic stability. The virus titers and RNA copy numbers over the passages fluctuated up to 1000-fold (Figure S3), with the largest changes in virus titers between passages three, four, and five. The consistent expression of Rluc over all 10 passages demonstrated the genetic stability of the r3MOPV (Figure S3). The ZsG expression was controlled through fluorescence microscopy. NGS of the P2, P5, and P10 viruses revealed four mutations (see Supplementary Table S1).

#### Growth Characteristics of r3MOPV

**Figure 2:**
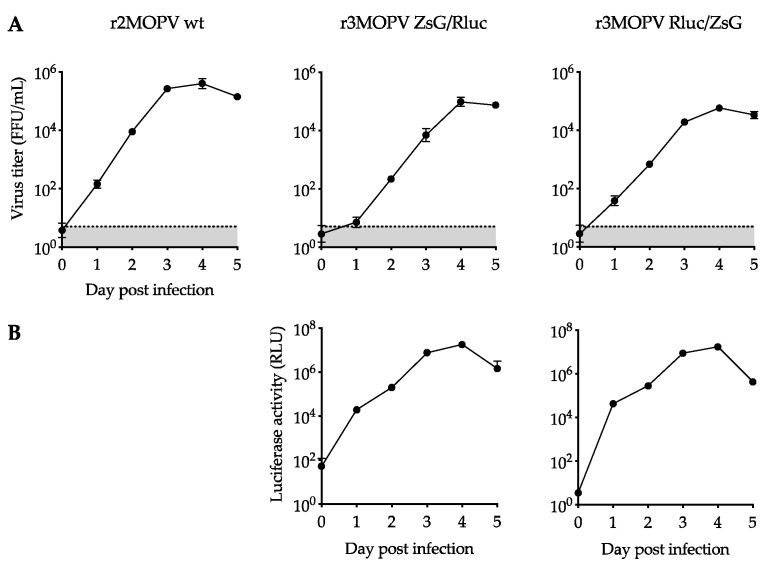
Growth kinetics of trisegmented rMOPV. Vero FM cells were infected with a MOI of 0.01 with r2MOPV wt, r3MOPV ZsG/Rluc, and r3MOPV Rluc/ZsG, and the virus replication was followed over 5 days. Virus titers were determined by immunofocus assay (**A**). Luciferase activity was measured for the two r3MOPV viruses (**B**). Plotted are the mean of three replicates and the standard deviation. The limit of detection for the titration is marked by a dashed line.

**Figure 3:**
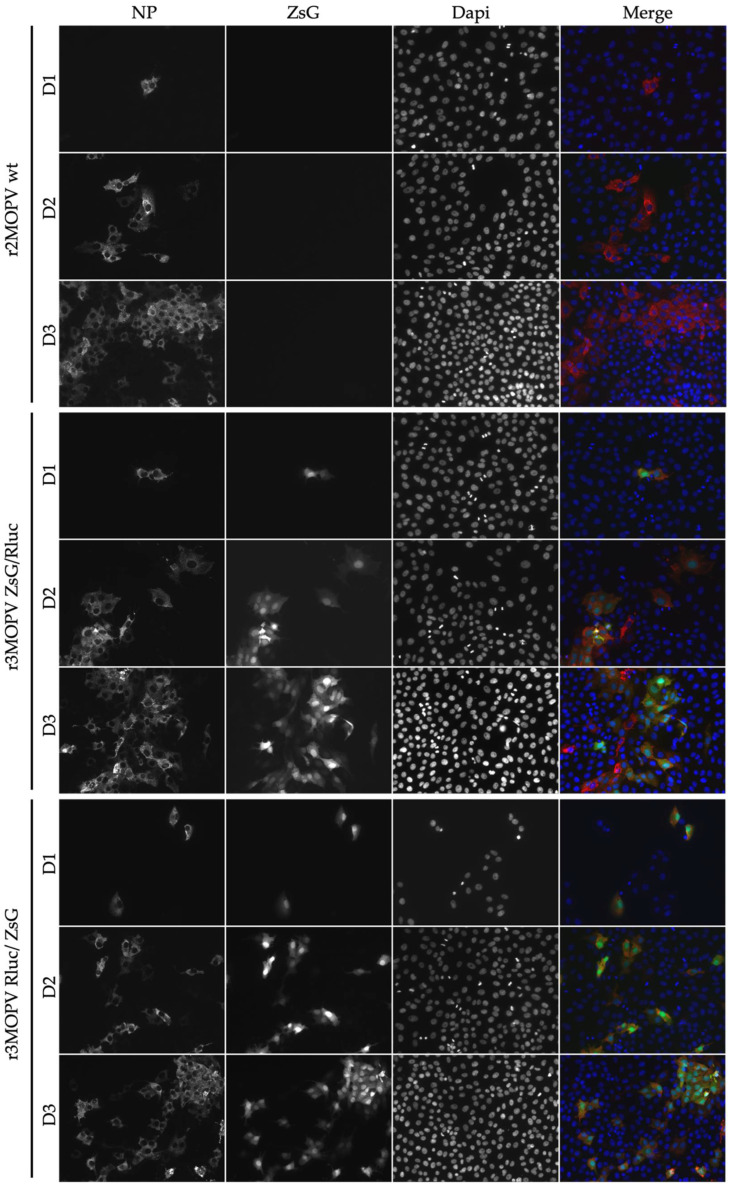
Immunofluorescence microscopy of r3MOPV. Vero FM cells were seeded on glass slides and infected with a MOI of 0.01 with r2MOPV wt, r3MOPV ZsG/Rluc, and r3MOPV Rluc/ZsG. The cells were fixed on days 1, 2, and 3 post infection and stained for NP (red). The nucleus was stained with Dapi (blue). ZsG is depicted in green.

The growth characteristics of the two r3MOPV viruses were compared with r2MOPV wt. Vero FM cells were infected with a MOI of 0.01 of the P1 stocks of the different viruses and virus titers (Figure 2A), Rluc activity (Figure 2B), and ZsG expression (Figure 3) were determined and compared.

The exponential growth of the viruses, seen through an increase of viral titers and luciferase activity between days one and three was observed. Both r3MOPV viruses grew slightly slower than the r2MOPV wt, with virus titers being 10–40-fold lower on days 2 and 3 post infection. Peak titers for all three viruses on day four post infection were similar, however. Similar to virus titer, luciferase activity peaked on day four and decreased slightly on day five. 

For the immunofluorescence microscopy, cells were seeded on glass slides and infected with the r2MOPV wt and the two r3MOPV. Cells were stained for NP and the nucleus was visualized with Dapi (Figure 3).

Virus spread, indicated by the number of NP-positive cells, was similar for all three viruses. ZsG expression could be observed for both r3MOPVs from day one post infection and reporter gene expression increased until day three. The expression of ZsG was similar for both r3MOPVs, independent of whether ZsG was in the NP or GP ORF.

### 3.3. Screening of Antiviral Compounds

**Figure 4:**
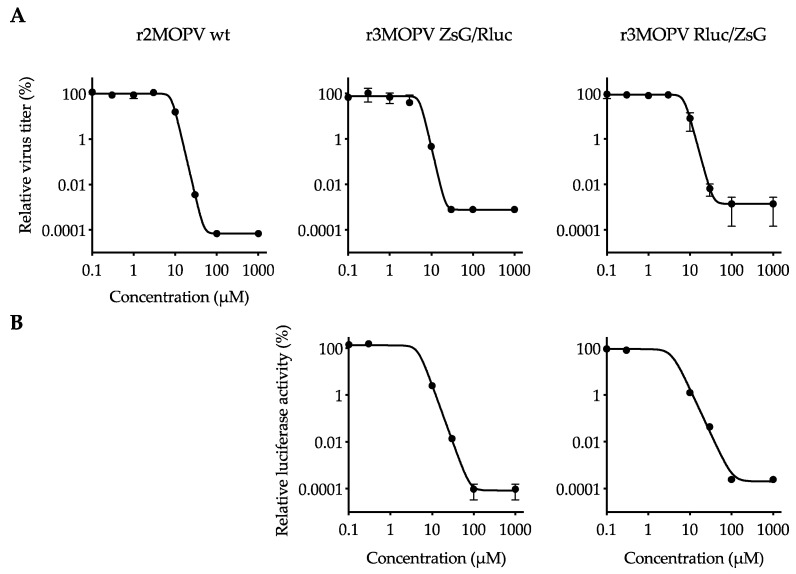
Antiviral activity of Ribavirin against MOPV. Vero FM cells were infected with r2MOPV wt, r3MOPV ZsG/Rluc, or r3MOPV Rluc/ZsG with a MOI of 0.01. Different concentrations of Ribavirin were added 1 h post infection. After 3 days, the concentration of infectious viral particles in the cell culture supernatant was measured by immunofocus assay (**A**). Cells were lysed and luciferase activity was determined (**B**). Sigmoidal dose–response curves were fitted to the data using Prism GraphPad 9. Shown are the mean and standard deviation of a representative experiment.

To evaluate the suitability of the r3MOPV viruses as a tool for easy screening of antiviral compounds, proof-of-principle virus inhibition experiments with the well-characterized compounds Favipiravir and Ribavirin were performed. Infected cells were incubated with a descending concentration of both compounds, and 3 days post infection the supernatant was harvested for virus titration and the cells were used to determine luciferase activity. Five independent experiments were performed, and representative plots for Ribavirin and Favipiravir are shown in Figure 4 and Supplementary Figure S4, respectively.

IC_50_ values for Favipiravir and Ribavirin were calculated for the individual experiments. They were similar for all three viruses within one experiment, and in the mean, less than a factor 2 difference between r2MOPV wt and the r3MOPVs was observed. Similarly, the comparison of virus titer and luciferase measurement-based IC_50_ values also showed less than a factor 2 difference within one experiment. IC_50_ values obtained in the different experiments showed a stronger fluctuation and varied up to factor 10 (Table 2).

**Figure 5:**
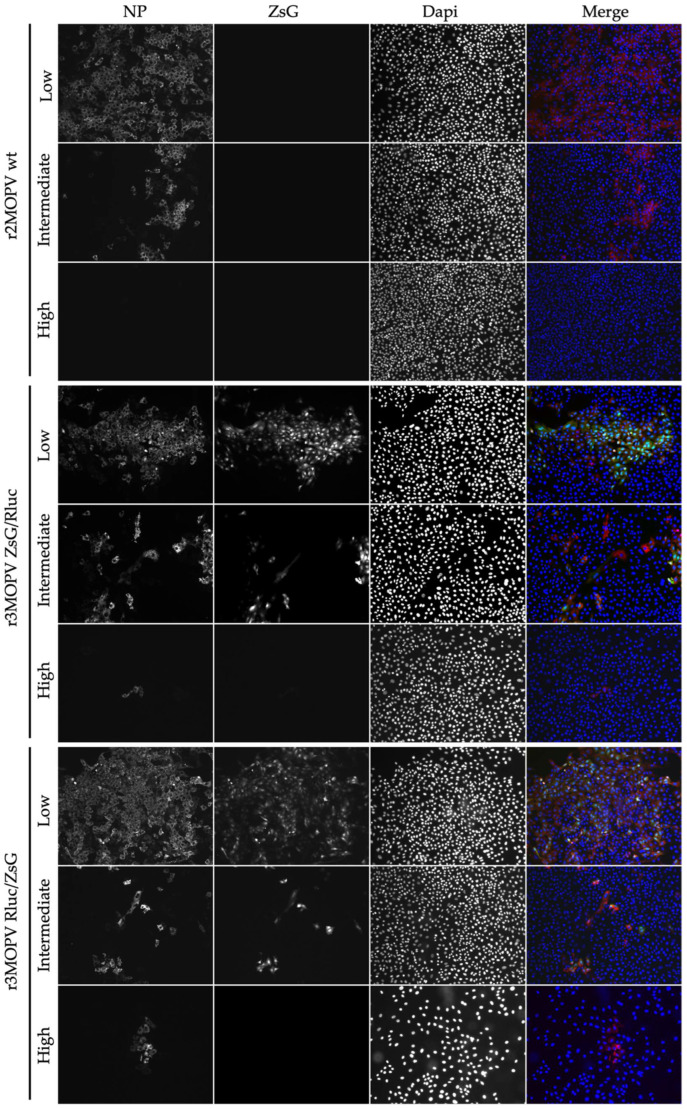
Antiviral activity of Ribavirin against MOPV. Vero FM cells were infected with r2MOPV wt, r3MOPV ZsG/Rluc, or r3MOPV Rluc/ZsG with a MOI of 0.01. Different concentrations of Ribavirin (low: 1 µM, intermediate: 10 µM, high 30 µM) were added 1 h post infection. Cells were fixed 3 days post infection and stained for NP (red) and Dapi (blue). ZsG is depicted in green.

For the evaluation of ZsG expression as a measure of antiviral activity of a compound, cells were infected and incubated with low (<½ IC_50_), intermediate (≈IC_50_), and high (>2 × IC_50_) concentrations of Ribavirin and Favipiravir. The cells were fixed and stained 3 days post infection (Figure 5 and Supplementary Figure S5). The cells incubated with the low drug concentrations had strong NP signals for all three viruses, and a good overlap of NP and the ZsG signals for the two r3MOPVs. Approximately the same number of infected cells could be detected by either ZsG or NP fluorescence. The infection rate decreased as expected with increasing drug concentrations. At the intermediate concentration, the number of infected cells was reduced by >50% compared to the low dose. The high drug dose reduced replication by more than 99%. ZsG and NP expression levels within infected cells were similar for all concentrations. Especially at intermediate concentrations, infected cells could easily be identified, and drug effects were restricted to reducing the number of infected cells.

## 4. Discussion

To rapidly identify effective broad-spectrum antiviral compounds against mammarenaviruses, high-throughput screening applications are essential. Large libraries of FDA-approved or experimental compounds are available. Using wild type viruses and classical read-outs of virus replication, these HTS applications are time-consuming. Thus, reporter gene-expressing virus systems, that reflect all viral “life cycle stages”, are powerful tools to rapidly screen and identify antiviral candidates. For arenaviruses some recombinant virus systems have already been established, including systems for LCMV [25,27,34,41,42], LASV [43,44], TACV [22,45], JUNV [19,46], PICV [29,47,48], and LUJV [20,49]. However, to date, no reverse genetic system for reporter-gene expressing MOPV has been described. To determine the most efficient RNA polymerase promoter system for MOPV, bisegmented recombinant MOPV variants were generated using S and L segment plasmids under the control of a T7 RNA polymerase or an RNA polymerase-I. In addition, the influence of S and L genome orientation (sense or antisense) was evaluated in the two polymerase systems. Consistent with results obtained with other mammarenaviruses, the rescue of rMOPV was successful in both systems [27,34,41]. However, virus rescue was more efficient using RNA Pol-I/II, irrespective of genomic orientation. As polymerases I and II are expressed in all mammalian cell lines, Pol-I/II-based systems are favorable and versatile with regard to the cell lines used for virus rescues. The T7-system in contrast needs the expression of T7 RNA polymerase, which requires the handling of transgenic T7 cell lines such as BSR-T7/5, or the co-transfection of T7 plasmids [27].

In addition to wild type-like recombinant viruses, the successful generation of several negative-strand RNA viruses expressing genes of interest has been demonstrated. Recombinant virus systems that express GFP or ZsG from a bicistronic NP-P2A-GFP/ZsG mRNA using the self-cleaving P2A peptide sequence have been described for LCMV, LASV, and LUJV [20,21,23]. Similar to a recombinant bicistronic Influenza virus containing an IRES, we also successfully rescued and characterized a bicistronic r2MOPV Rluc with a GP-IRES-Rluc cassette. However, this virus was genetically unstable. The loss of the reporter gene Rluc after two passages made r2MOPV Rluc an unsuitable candidate for HTS. In contrast, we observed high genetic stability and integrity in our trisegmented r3MOPV variants (r3MOPV ZsG/Rluc and r3MOPV Rluc/ZsG). These findings are in line with reports for other Bunyaviruses [22,25,26,28,29]. Moreover, both r3MOPVs showed continuous reporter gene expression, had comparable growth characteristics over several virus passages, and only slightly lower maximum titers compared to the r2MOPV wt. Thus, expression levels of the two easily measured reporter genes served as a valid surrogate for virus amplification in infected cells. The suitability of both r3MOPVs for HTS antiviral screening was evaluated in proof-of-principle virus inhibition assays with the well-established anti-arenaviral drugs Favipiravir and Ribavirin. Microscopy analyses of r2MOPV wt and r3MOPV infected cells treated with Favipiravir or Ribavirin revealed comparable antiviral effects on all three viruses. This shows that in our system ZsG is a suitable readout of fluorescence microscopy-based measurement of antiviral effects. Even though the number of ZsG-positive cells was not quantified in this study, sophisticated state-of-the-art fluorescent microscopes with automated picture acquisition and quantification software would allow for a high-throughput evaluation of antiviral compounds based on ZsG-expression. A similar approach has for example been successfully used to identify novel compounds that are active against LASV or LUJV [20,24].

Notably, we obtained comparable IC_50_ values by analyzing luciferase activities and virus titration (intraassay IC_50_ variation of less than factor 2). IC_50_ values were also in the same range for the three viruses, indicating that the r3MOPVs had similar sensitivity to the tested drugs. Our data shows that Rluc measurement is a good and quantitative surrogate for virus titer determination and allows easy evaluation of drug effects. The Rluc values for the positive and negative controls differed by more than 5 log, which is a good dynamic range for an antiviral assay. We had a Z factor of 0.68 for the Rluc-based readout, indicating that our assay is suitable for HTS [50]. It should be mentioned that both drugs have a similar mode of action and inhibit virus replication. The suitability of this system to screen for entry inhibitors, for instance, would need to be validated in the future. In our system with a non-secreted luciferase, several hands-on steps were needed to prepare the samples for measurement, which could be a disadvantage for HTS. Replacing Rluc with a secreted luciferase [51,52] would further enhance the suitability of the r3MOPVs for large-scale screening of antiviral compounds. In addition, it would allow for the parallel determination of virus infection and/or of cytotoxic effects of compounds on the cells by classical cell-viability assays. In conclusion, this study describes the generation and characterization of recombinant Mopeia viruses with trisegmented genomes stably expressing the reporter genes Rluc and ZsG. Reporter gene expression can be easily quantified, and provides an accurate surrogate of the viral titer. Thus, our reporter viruses may be considered direct candidates for HTS applications in BSL-2 containment, aimed at identifying arenavirus inhibitors.

## Figures and Tables

**Table 1:** Summary of rescue success for the r2MOPV wt. A rescue was successful if an infectious virus was recovered within three weeks post transfection. The number of successfully rescued viruses is given with the total number of transfections in brackets.

Genome Orientation	Promotor	Number of Rescued Viruses and Transfections
Genomic	T7	*n* = 0 (out of >10)
Antigenomic	T7	*n* = 1 (out of >10)
Genomic	Pol-I/II	*n* = 4 (out of 5)
Antigenomic	Pol-I/II	*n* = 8 (out of 10)

**Table 2:** Comparison of IC_50_ values determined through 4PL sigmoidal fitting of virus titer reduction or luciferase activity reduction after Favipiravir and Ribavirin treatment. Mean and range of five independent experiments.

		IC_50_ Favipiravir (µM)	IC_50_ Ribavirin (µM)
Virus titer	r2MOPV wt	25.7 (20.5–31.8)	15.5 (6.6–36.8)
	r3MOPV ZsG/Rluc	17.8 (6.5–28.2)	13.6 (5.2–38.9)
	r3MOPV Rluc/ZsG	25.3 (14.6–31.8)	26.4 (7.1–38.1)
Rluc	r2MOPV wt	/	/
	r3MOPV ZsG/Rluc	32.5 (7.4–35.8)	13.6 (3.4–41.7)
	r3MOPV Rluc/ZsG	33.0 (8.8–70.2)	17.1 (3.6–34.0)

## Data Availability

Not applicable.

## Supplementary Materials

The following supporting information can be downloaded at: https://www.mdpi.com/article/10.3390/v14091869/s1, Figure S1. Growth kinetics of MOPV wt and r2MOPV wt; Figure S2. Growth kinetics of bisegmented rMOPV; Figure S3. Stability evaluation of r3MOPV; Figure S4. Antiviral activity of Favipiravir against MOPV; Figure S5. Antiviral activity of Favipiravir against rMOPV; Table S1: Mutations in coding regions after serial passaging of r2MOPV wt and r3MOPVs.

## References

[1] Nichol T.S., Arikawa J., Kawaoka Y. (2000). Emerging viral diseases. Proc. Natl. Acad. Sci. USA.

[2] Wolfe D.N., Dunavan C.P., Diamond J. (2007). Origins of major human infectious diseases. Nature.

[3] Koundouno F.R., Kafetzopoulou L.E., Faye M., Renevey A., Soropogui B., Ifono K., Nelson E.V., Kamano A.A., Tolno C., Annibaldis G. (2022). Detection of Marburg Virus Disease in Guinea. N. Engl. J. Med..

[4] Keita A.K., Koundouno F.R., Faye M., Düx A., Hinzmann J., Diallo H., Ayouba A., Le Marcis F., Soropogui B., Ifono K. (2021). Resurgence of Ebola virus in 2021 in Guinea suggests a new paradigm for outbreaks. Nature.

[5] Makoni M. (2022). Ebola outbreak in DR Congo. Lancet.

[6] Mehand M.S., Al-Shorbaji F., Millett P., Murgue B. (2018). The WHO R&D Blueprint: 2018 review of emerging infectious diseases requiring urgent research and development efforts. Antivir. Res..

[7] Maes P., Alkhovsky S.V., Bào Y., Beer M., Birkhead M., Briese T., Buchmeier M.J., Calisher C.H., Charrel R.N., Choi I.R. (2018). Taxonomy of the family Arenaviridae and the order Bunyavirales: Update 2018. Arch. Virol..

[8] Brisse E.M., Ly H. (2019). Hemorrhagic Fever-Causing Arenaviruses: Lethal Pathogens and Potent Immune Suppressors. Front. Immunol..

[9] Fichet-Calvet E., Rogers D.J. (2009). Risk maps of Lassa fever in West Africa. PLoS Negl. Trop. Dis..

[10] McCormick J.B., Webb P.A., Krebs J.W., Johnson K.M., Smith E.S. (1987). A prospective study of the epidemiology and ecology of Lassa fever. J. Infect. Dis..

[11] McCormick J.B., King I.J., Webb P.A., Scribner C.L., Craven R.B., Johnson K.M., Elliott L.H., Belmont-Williams R. (1986). Lassa fever. Effective therapy with ribavirin. N. Engl. J. Med..

[12] Eberhardt K.A., Mischlinger J., Jordan S., Groger M., Günther S., Ramharter M. (2019). Ribavirin for the treatment of Lassa fever: A systematic review and meta-analysis. Int. J. Infect. Dis..

[13] Salam A.P., Cheng V., Edwards T., Olliaro P., Sterne J., Horby P. (2021). Time to reconsider the role of ribavirin in Lassa fever. PLoS Negl. Trop. Dis..

[14] Hass M., Gölnitz U., Müller S., Becker-Ziaja B., Günther S. (2004). Replicon system for Lassa virus. J. Virol..

[15] Lee K.J., Novella I.S., Teng M.N., Oldstone M.B.A., de la Torre J.C. (2000). NP and L proteins of lymphocytic choriomeningitis virus (LCMV) are sufficient for efficient transcription and replication of LCMV genomic RNA analogs. J. Virol..

[16] Lopez N., Jacamo R., Franze-Fernandez M.T. (2001). Transcription and RNA replication of tacaribe virus genome and antigenome analogs require N and L proteins: Z protein is an inhibitor of these processes. J. Virol..

[17] Auperin D.D., Romanowski V., Galinski M., Bishop D.H. (1984). Sequencing studies of pichinde arenavirus S RNA indicate a novel coding strategy, an ambisense viral S RNA. J. Virol..

[18] Gunther S., Lenz O. (2004). Lassa virus. Crit. Rev. Clin. Lab. Sci..

[19] McKee K.T., Oro J.G.B., Kuehne A.I., Spisso J.A., Mahlandt B.G. (1992). Candid No. 1 Argentine hemorrhagic fever vaccine protects against lethal Junin virus challenge in rhesus macaques. Intervirology.

[20] Welch S.R., Spengler J., Genzer S., Chatterjee P., Flint M., Bergeron ., Montgomery J., Nichol S., Albariño C., Spiropoulou C. (2021). Screening and Identification of Lujo Virus Inhibitors Using a Recombinant Reporter Virus Platform. Viruses.

[21] Cai Y., Iwasaki M., Beitzel B.F., Yú S., Postnikova E.N., Cubitt B., DeWald L.E., Radoshitzky S.R., Bollinger L., Jahrling P.B. (2018). Recombinant Lassa Virus Expressing Green Fluorescent Protein as a Tool for High-Throughput Drug Screens and Neutralizing Antibody Assays. Viruses.

[22] Ye C., de la Torre J.C., Martinez-Sobrido L. (2020). Development of Reverse Genetics for the Prototype New World Mammarenavirus Tacaribe Virus. J. Virol..

[23] Ngo N., Cubitt B., Iwasaki M., de la Torre J.C. (2015). Identification and Mechanism of Action of a Novel Small-Molecule Inhibitor of Arenavirus Multiplication. J. Virol..

[24] Welch S.R., Guerrero L.W., Chakrabarti A.K., McMullan L., Flint M., Bluemling G.R., Painter G.R., Nichol S.T., Spiropoulou C.F., Albariño C.G. (2016). Lassa and Ebola virus inhibitors identified using minigenome and recombinant virus reporter systems. Antivir. Res..

[25] Emonet S.F., Garidou L., McGavern D.B., de la Torre J.C. (2009). Generation of recombinant lymphocytic choriomeningitis viruses with trisegmented genomes stably expressing two additional genes of interest. Proc. Natl. Acad. Sci. USA.

[26] Cheng B.Y., Ortiz-Riaño E., de la Torre J.C., Martínez-Sobrido L. (2015). Arenavirus Genome Rearrangement for the Development of Live Attenuated Vaccines. J. Virol..

[27] Ortiz-Riano E., Cheng B.Y.H., de la Torre J.C., Martínez-Sobrido L. (2013). Arenavirus reverse genetics for vaccine development. J. Gen. Virol..

[28] Dhanwani R., Ly H., Liang Y. (2017). Recombinant Tri-Segmented Pichinde Virus as a Novel Live Viral Vaccine Platform. Methods Mol. Biol..

[29] Dhanwani R., Zhou Y., Huang Q., Verma V., Dileepan M., Ly H., Liang Y. (2015). A Novel Live Pichinde Virus-Based Vaccine Vector Induces Enhanced Humoral and Cellular Immunity after a Booster Dose. J. Virol..

[30] Pfefferle S., Krähling V., Ditt V., Grywna K., Mühlberger E., Drosten C. (2009). Reverse genetic characterization of the natural genomic deletion in SARS-Coronavirus strain Frankfurt-1 open reading frame 7b reveals an attenuating function of the 7b protein in-vitro and in-vivo. Virol. J..

[31] Kummerer M.B., Rice C.M. (2002). Mutations in the yellow fever virus nonstructural protein NS2A selectively block production of infectious particles. J. Virol..

[32] Buchholz J.U., Finke S., Conzelmann K.K. (1999). Conzelmann, Generation of bovine respiratory syncytial virus (BRSV) from cDNA: BRSV NS2 is not essential for virus replication in tissue culture, and the human RSV leader region acts as a functional BRSV genome promoter. J. Virol.

[33] Kerber R., Rieger T., Busch C., Flatz L., Pinschewer D.D., Kümmerer B.M., Günther S. (2011). Cross-species analysis of the replication complex of Old World arenaviruses reveals two nucleoprotein sites involved in L protein function. J. Virol..

[34] Flatz L., Bergthaler A., de la Torre J.C., Pinschewer D.D. (2006). Recovery of an arenavirus entirely from RNA polymerase I/II-driven cDNA. Proc. Natl. Acad. Sci. USA.

[35] Niwa H., Yamamura K., Miyazaki J. (1991). Efficient selection for high-expression transfectants with a novel eukaryotic vector. Gene.

[36] Pinschewer D.D., Perez M., Sanchez A.B., de la Torre J.C. (2003). Recombinant lymphocytic choriomeningitis virus expressing vesicular stomatitis virus glycoprotein. Proc. Natl. Acad. Sci. USA.

[37] Rieger T., Merkler D., Gunther S. (2013). Infection of type I interferon receptor-deficient mice with various old world arenaviruses: A model for studying virulence and host species barriers. PLoS ONE.

[38] Gunther S. (2000). Imported lassa fever in Germany: Molecular characterization of a new lassa virus strain. Emerg. Infect. Dis..

[39] Hufert T.F., Ludke W., Schmitz H. (1989). Epitope mapping of the Lassa virus nucleoprotein using monoclonal anti-nucleocapsid antibodies. Arch. Virol..

[40] Cadar D., Jellinger K.A., Riederer P., Strobel S., Monoranu C.M., Tappe D. (2021). No Metagenomic Evidence of Causative Viral Pathogens in Postencephalitic Parkinsonism Following Encephalitis Lethargica. Microorganisms.

[41] Sanchez A.B., de la Torre J.C. (2006). Rescue of the prototypic Arenavirus LCMV entirely from plasmid. Virology.

[42] Bonilla W.V., Kirchhammer N., Marx A.-F., Kallert S.M., Krzyzaniak M.A., Lu M., Darbre S., Schmidt S., Raguz J., Berka U. (2021). Heterologous arenavirus vector prime-boost overrules self-tolerance for efficient tumor-specific CD8 T cell attack. Cell Rep. Med..

[43] Albarino C.G., Bird B.H., Chakrabarti A.K., Dodd K.A., Erickson B.R., Nichol S.T. (2011). Efficient rescue of recombinant Lassa virus reveals the influence of S segment noncoding regions on virus replication and virulence. J. Virol..

[44] Welch S.R., Scholte F., Albariño C.G., Kainulainen M.H., Coleman-McCray J.D., Guerrero L.W., Chakrabarti A.K., Klena J.D., Nichol S.T., Spengler J.R. (2018). The S Genome Segment Is Sufficient to Maintain Pathogenicity in Intra-Clade Lassa Virus Reassortants in a Guinea Pig Model. Front. Cell. Infect. Microbiol..

[45] Foscaldi S., Loureiro M.E., Sepúlveda C., Palacios C., Forlenza M.B., López N. (2020). Development of a Reverse Genetic System to Generate Recombinant Chimeric Tacaribe Virus that Expresses Junin Virus Glycoproteins. Pathogens.

[46] Emonet S.F., Seregin A.V., Yun N.E., Poussard A.L., Walker A.G., de la Torre J.C., Paessler S. (2011). Rescue from cloned cDNAs and in vivo characterization of recombinant pathogenic Romero and live-attenuated Candid #1 strains of Junin virus, the causative agent of Argentine hemorrhagic fever disease. J. Virol..

[47] Kumar P., Sharafeldin T.A., Kumar R., Huang Q., Liang Y., Goyal S.M., Porter R.E., Ly H., Mor S.K. (2021). Development of a Recombinant Pichinde Virus-Vectored Vaccine against Turkey Arthritis Reovirus and Its Immunological Response Characterization in Vaccinated Animals. Pathogens.

[48] Lan S., McLay Schelde L., Wang J., Kumar N., Ly H., Liang Y. (2009). Development of infectious clones for virulent and avirulent pichinde viruses: A model virus to study arenavirus-induced hemorrhagic fevers. J. Virol..

[49] Bergeron E., Chakrabarti A.K., Bird B.H., Dodd K.A., McMullan L.K., Spiropoulou C.F., Nichol S.T., Albariño C.G. (2012). Reverse genetics recovery of Lujo virus and role of virus RNA secondary structures in efficient virus growth. J. Virol..

[50] Zhang H.J., Chung T.D., Oldenburg K.R. (1999). A Simple Statistical Parameter for Use in Evaluation and Validation of High. Throughput Screening Assays. J. Biomol. Screen..

[51] Falk J.J., Sampaio K.L., Stegmann C., Lieber D., Kropff B., Mach M., Sinzger C. (2016). Generation of a Gaussia luciferase-expressing endotheliotropic cytomegalovirus for screening approaches and mutant analyses. J. Virol. Methods.

[52] Eckert N., Wrensch F., Gärtner S., Palanisamy N., Goedecke U., Jäger N., Pöhlmann S., Winkler M. (2014). Influenza A virus encoding secreted Gaussia luciferase as useful tool to analyze viral replication and its inhibition by antiviral compounds and cellular proteins. PLoS ONE.

