# Supplementary material for: Establishment of Recombinant Trisegmented Mopeia Virus Expressing Two Reporter Genes for Screening of Mammarenavirus Inhibitors
Source: Viruses. 2022 Aug 25;14(9):1869. doi: 10.3390/v14091869 (PMC9505675; doi:10.3390/v14091869)
Supplement: Supplementary file 1 [file viruses-14-01869-s001.zip › viruses-1867481-supplementary.pdf]

Table S1: Mutations in coding regions after serial passaging of r2MOPV wt and r3MOPVs.

Virus	Gene	Passage	Nucleotide exchange	Amino acid exchange
r3MOPV ZsG/Rluc	GPC	From P2	Nucleotide 575: AGA → AAA	Amino acid 192: Arg → Lys
		From P10	Nucleotide 445: TAT → CAT	Amino acid 149: Tyr → His
	RLuc	From P5	Nucleotide 923: AAG → AGG	Amino acid 308: Lys → Arg
r3MOPV Rluc/ZsG	GPC	From P5	Nucleotide 445: TAT → CAT	Amino acid 149: Tyr → His

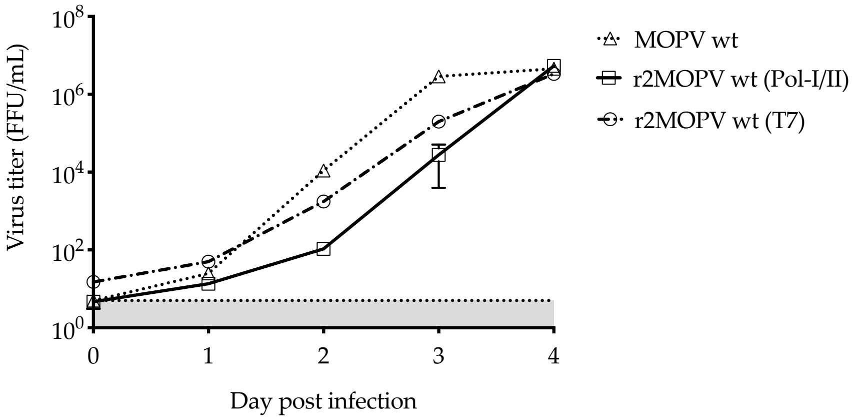

Figure S1. Growth kinetics of MOPV wt and r2MOPV wt. Vero FM cells were infected with a MOI of 0.01 with r2MOPV wt rescued in the Pol-I/II or the T7 polymerase system (both from antigenomic orientation of the segments), and MOPV wt and the virus replication was followed over 4 days. Virus titers were determined by immunofocus assay. Plotted are the mean of three replicates and the standard deviation. The limit of detection for the assays are marked by a dashed line.

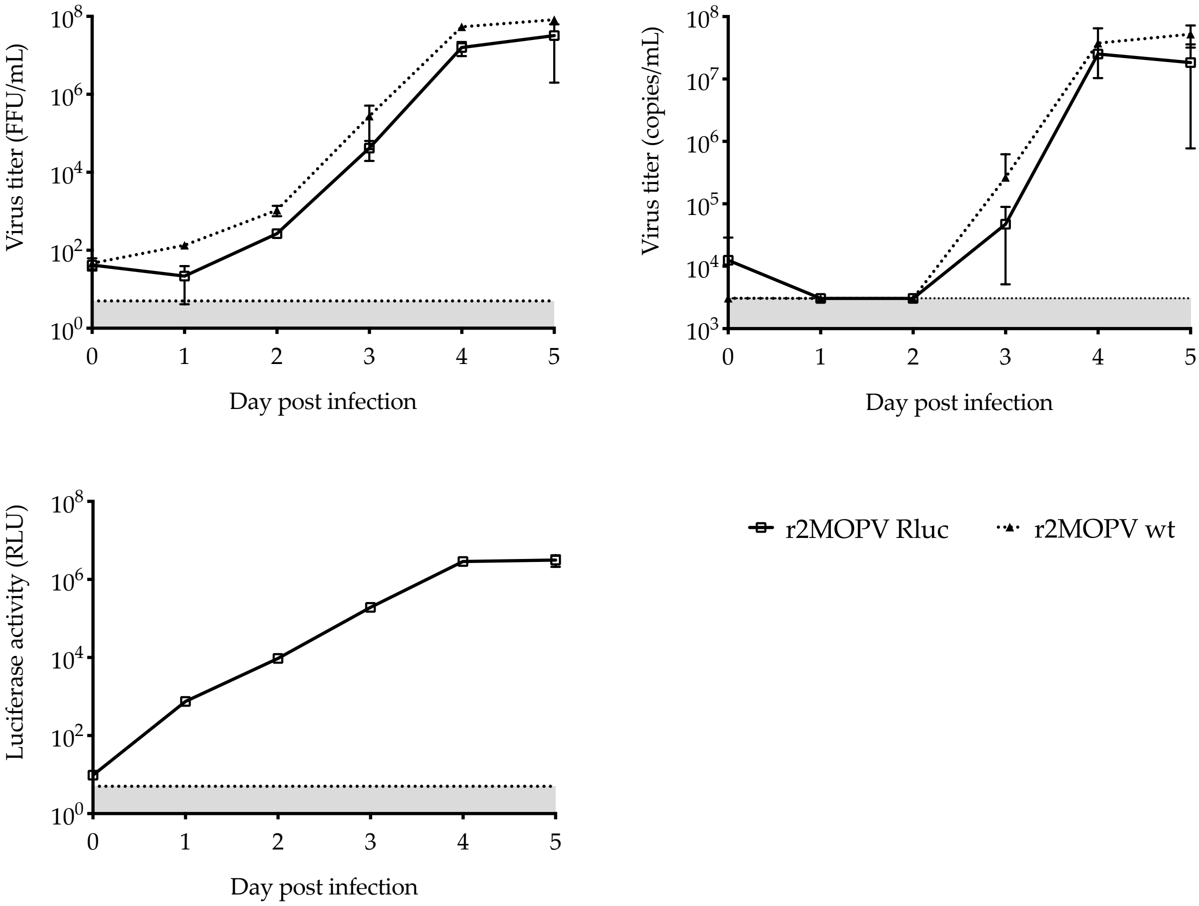

Figure S2. Growth kinetics of bisegmented rMOPV. Vero FM cells were infected with a MOI of 0.01 with r2MOPV wt and r2MOPV Rluc, and the virus replication was followed over 5 days. Virus titers were determined by immunofocus assay and RT-PCR. Luciferase activity was measured for the r2MOPV Rluc virus. Plotted are the mean of three replicates and the standard deviation. The limit of detection for the assays are marked by a dashed line.

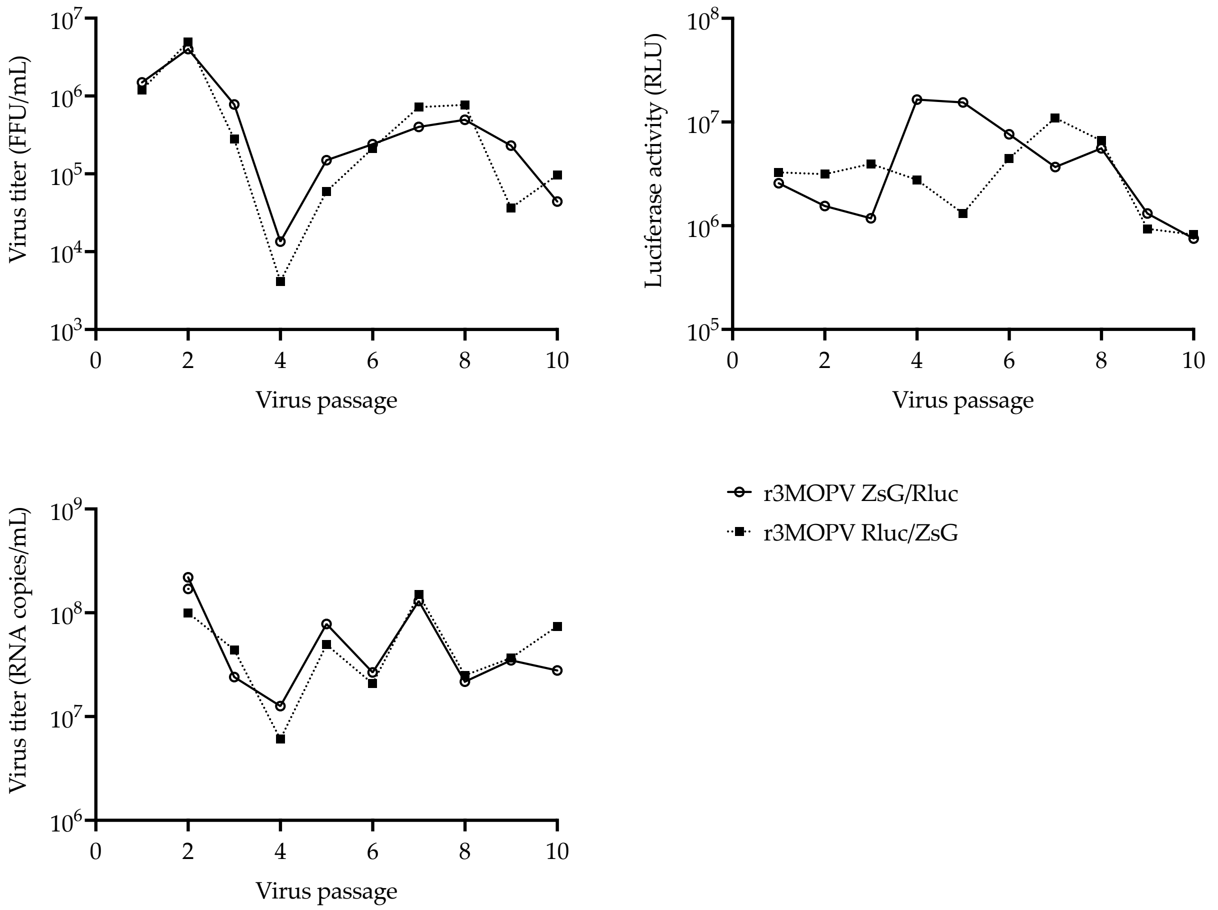

Figure S3. Stability evaluation of r3MOPV. r3MOPV ZsG/Rluc and r3MOPV Rluc/ZsG were passaged on Vero FM cells. Every passage virus titer, RNA copy numbers and Luciferase activity were determined.

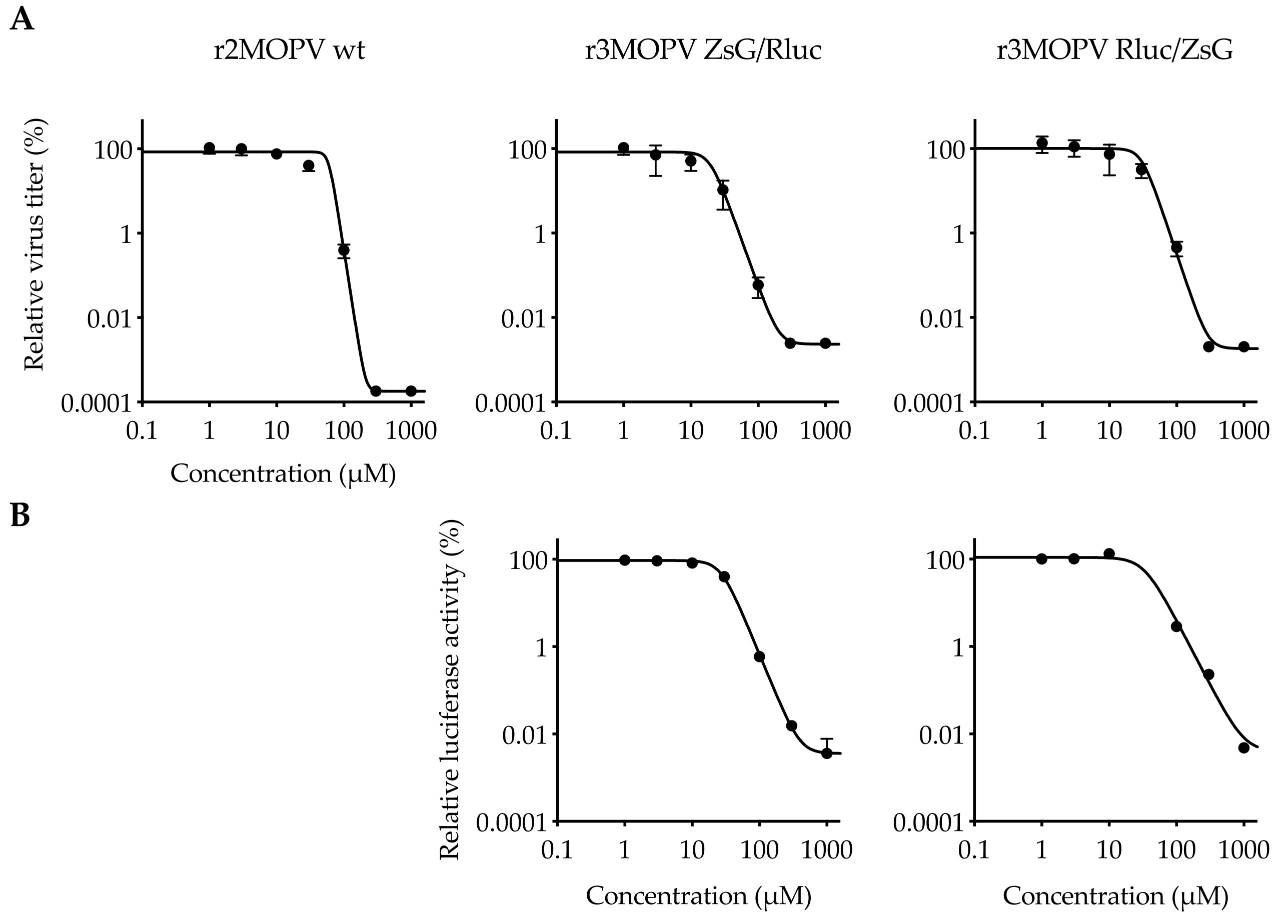

Figure S4. Antiviral activity of Favipiravir against MOPV. Vero FM cells were infected with r2MOPV wt, r3MOPV ZsG/Rluc, or r3MOPV Rluc/ZsG with a MOI of 0.01. Different concentrations of Favipiravir were added 1 h post infection. After 3 days, the concentration of infectious viral particles in the cell culture supernatant was measured by immunofocus assay (A). Cells were lysed and luciferase activity was determined (B). Sigmoidal dose–response curves were fitted to the data using Prism GraphPad 9. Shown are the mean and standard deviation of a representative experiment.

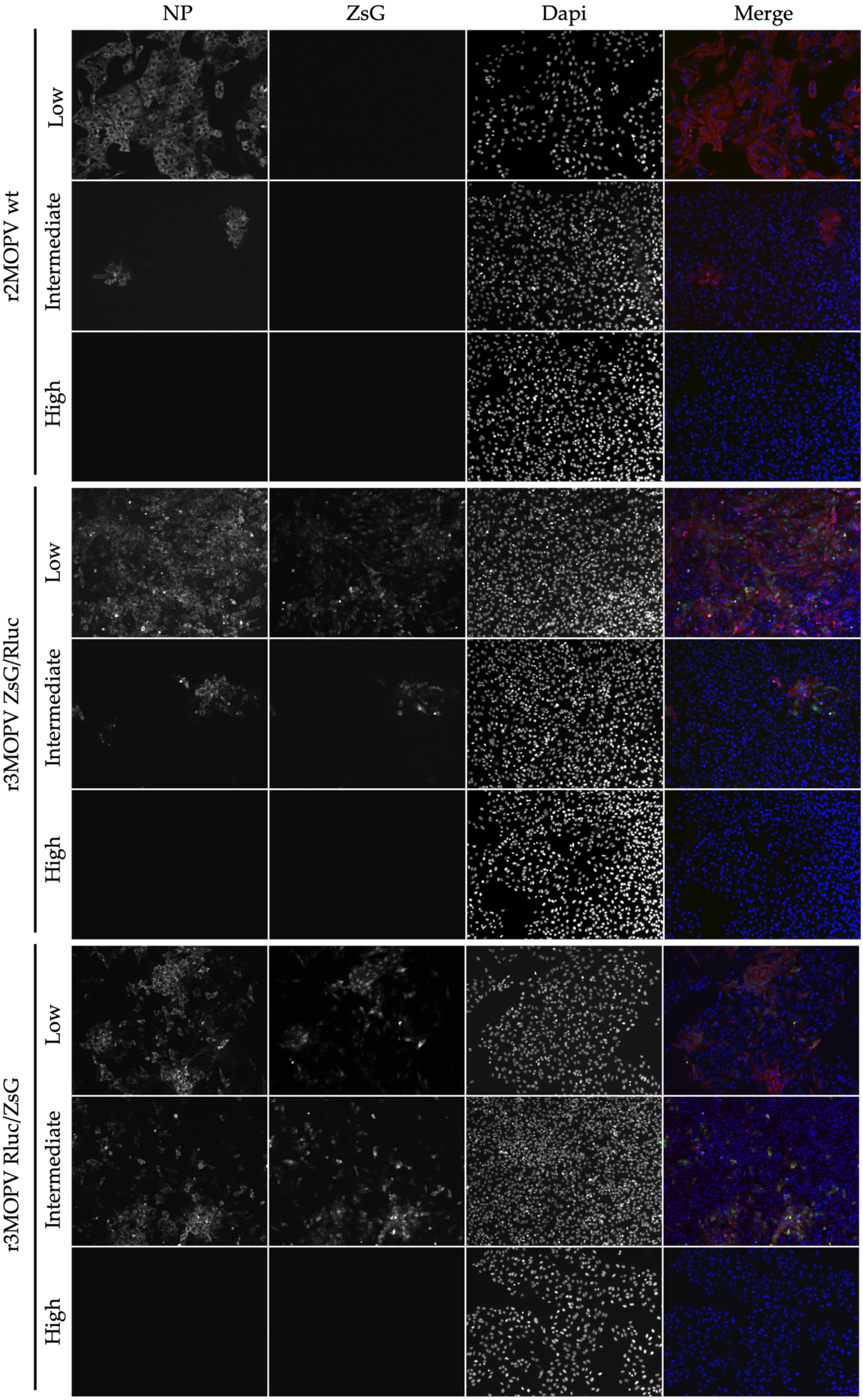
NP
ZsG
Dapi
Merge
r2MOPV wt
Low
Intermediate
High
r3MOPV ZsG/Rluc
Low
Intermediate
High
r3MOPV Rluc/ZsG
Low
Intermediate
High

Figure S5. Antiviral activity of Favipiravir against rMOPV. Vero FM cells were infected with r2MOPV wt, r3MOPV ZsG/Rluc, or r3MOPV Rluc/ZsG with a MOI of 0.01. Different concentrations of Favipiravir (low: 3 μM, intermediate: 30 μM, high: 100 μM) were added 1 h post infection. Cells were fixed 3 days post infection and stained for NP (red) and Dapi (blue). ZsG is depicted in green.
